# Supplementary material for: Multi‐dataset identification of innovative feature genes and molecular mechanisms in keratoconus
Source: J Cell Mol Med. 2024 Sep 19;28(18):e70079. doi: 10.1111/jcmm.70079 (PMC11412914; doi:10.1111/jcmm.70079)
Supplement: Supplementary file 2 — Material S2. [file JCMM-28-e70079-s002.zip › 7.GSEA analysis/3.DBNDD1/KEGG_RNA_POLYMERASE.html]

Details for gene set KEGG\_RNA\_POLYMERASE[GSEA]

|  || Dataset | DBNDD1.group.cls#h\_versus\_l |
| Phenotype | group.cls#h\_versus\_l |
| Upregulated in class | h |
| GeneSet | KEGG\_RNA\_POLYMERASE |
| Enrichment Score (ES) | 0.23353122 |
| Normalized Enrichment Score (NES) | 0.7573783 |
| Nominal p-value | 0.86247545 |
| FDR q-value | 1.0 |
| FWER p-Value | 1.0 |
Table: GSEA Results Summary

  

Fig 1: Enrichment plot: KEGG\_RNA\_POLYMERASE      
 Profile of the Running ES Score & Positions of GeneSet Members on the Rank Ordered List

  

| SYMBOL | TITLE | RANK IN GENE LIST | RANK METRIC SCORE | RUNNING ES | CORE ENRICHMENT || 1 | POLR1A | na | 6381 | 0.140 | -0.0373 | Yes |
| 2 | POLR2J2 | na | 8504 | 0.094 | 0.0007 | Yes |
| 3 | POLR3H | na | 9839 | 0.073 | 0.0393 | Yes |
| 4 | POLR3B | na | 10585 | 0.063 | 0.0844 | Yes |
| 5 | POLR1B | na | 11861 | 0.048 | 0.0984 | Yes |
| 6 | POLR2H | na | 13862 | 0.031 | 0.0735 | Yes |
| 7 | POLR2C | na | 14050 | 0.030 | 0.0991 | Yes |
| 8 | POLR2I | na | 14158 | 0.029 | 0.1262 | Yes |
| 9 | POLR1C | na | 14659 | 0.025 | 0.1383 | Yes |
| 10 | POLR2J3 | na | 14921 | 0.023 | 0.1553 | Yes |
| 11 | POLR2J | na | 15425 | 0.020 | 0.1615 | Yes |
| 12 | POLR3K | na | 15696 | 0.018 | 0.1725 | Yes |
| 13 | POLR2D | na | 16178 | 0.015 | 0.1739 | Yes |
| 14 | POLR3A | na | 16649 | 0.012 | 0.1725 | Yes |
| 15 | POLR2K | na | 17057 | 0.009 | 0.1702 | Yes |
| 16 | POLR3GL | na | 17941 | 0.003 | 0.1480 | Yes |
| 17 | POLR2A | na | 17989 | 0.003 | 0.1496 | Yes |
| 18 | POLR2G | na | 18012 | 0.003 | 0.1517 | Yes |
| 19 | POLR2B | na | 18643 | -0.002 | 0.1354 | Yes |
| 20 | POLR2L | na | 19025 | -0.005 | 0.1293 | Yes |
| 21 | POLR1D | na | 20038 | -0.013 | 0.1141 | Yes |
| 22 | POLR2E | na | 20180 | -0.015 | 0.1254 | Yes |
| 23 | POLR3F | na | 20850 | -0.021 | 0.1278 | Yes |
| 24 | POLR3D | na | 22729 | -0.040 | 0.1157 | Yes |
| 25 | POLR2F | na | 23137 | -0.045 | 0.1511 | Yes |
| 26 | POLR1E | na | 25248 | -0.074 | 0.1683 | Yes |
| 27 | POLR3G | na | 26624 | -0.100 | 0.2335 | Yes |
Table: GSEA details [plain text format]

  

Fig 2: KEGG\_RNA\_POLYMERASE      
 Blue-Pink O' Gram in the Space of the Analyzed GeneSet

  

Fig 3: KEGG\_RNA\_POLYMERASE: Random ES distribution      
 Gene set null distribution of ES for **KEGG\_RNA\_POLYMERASE**
